# The Impact of Co-occurring Anxiety and Alcohol Use Disorders on Video Telehealth Utilization Among Rural Veterans

**DOI:** 10.1007/s41347-020-00150-x

**Published:** 2020-08-06

**Authors:** Anthony H. Ecker, Amber B. Amspoker, Julianna B. Hogan, Jan A. Lindsay

**Affiliations:** 1 Houston VA HSR&D Center for Innovations in Quality, Effectiveness and Safety, Michael E. DeBakey VA Medical Center (MEDVAMC 152), 2002 Holcombe Blvd, Houston, TX 77030 USA; 2 Baylor College of Medicine, Houston, TX USA; 3 VA South Central Mental Illness Research, Education and Clinical Center (a virtual center), Houston, TX USA

**Keywords:** Anxiety disorders, Alcohol use disorder, Veterans, Telemedicine

## Abstract

Co-occurring anxiety and alcohol use disorders lead to poorer treatment outcomes for both disorders. Compounding risk for poor outcomes related to these disorders, individuals living in rural areas face barriers receiving evidence-based mental health treatment. Video to home telehealth (VTH) has been implemented broadly within the Veterans Health Administration to improve access to care for rural veterans. However, VTH may not be utilized equally across disorders and comorbidities, including co-occurring anxiety and alcohol use disorders, potentially contributing to gaps in care that are not available in person. A cohort of veterans who received at least one VTH mental health visit between fiscal years 2016–2019 was compiled from VA administrative data. Multilevel linear growth curve models were used to examine growth in VTH use over time among veterans with anxiety only, alcohol use disorder only, and co-occurring disorders. Fixed effects were significant for both time and diagnosis group and a significant interaction between time and group. For each subsequent fiscal year, the percentage of total MH visits that were VTH increased for all groups but less so for those with co-occurring anxiety and alcohol use diagnoses. Despite VTH being an important tool to reach underserved rural veterans, rural veterans with AUD and co-occurring anxiety and AUD are at risk for not receiving care using this modality. Findings suggest that veterans with co-occurring anxiety and AUD are especially at risk for being underserved, given that a major goal of VTH is to increase access to mental health services.

Anxiety disorders are among the most common psychiatric disorders, with a prevalence rate of 18% (Kessler et al. 2005). In a given year, one in four adults is at risk of meeting criteria for anxiety disorders (Beesdo et al. 2010). These conditions are characterized by intense fear and avoidance of a range of stimuli (e.g., social situations, worry, bodily sensations), resulting in high levels of social, occupational, relational, and physical distress and impairment (American Psychiatric Association 2013; Norton and Barrera 2012; Stein et al. 2005). Anxiety disorders commonly co-occur with alcohol use disorder (AUD), one of the most commonly used substances in the world. In a given year, 13% of individuals with AUD also meet criteria for an anxiety disorder (Grant et al. 2004). Importantly, these disorders are longitudinally linked, as individuals with anxiety disorders are more than seven times as likely to later develop an AUD as individuals without AUD (Buckner and Turner 2009); and anxiety disorders predict later AUD but not vice versa (Wolitzky-Taylor et al. 2012). Neurobiological elements, including circuits in the amygdala, influence feedback loops between negative affect (including anxiety) and alcohol, contributing to onset and maintenance of these co-occurring disorders (for reviews see Anker and Kushner 2019; Gilpin et al. 2015). Anxiety disorders and AUD are a pernicious combination that has a strong negative impact on individuals (Smith and Randall 2012).

Co-occurring anxiety and AUD are also related to poorer treatment outcomes for both disorders. Among individuals undergoing outpatient treatment for AUD, those with co-occurring anxiety disorders reported greater alcohol consumption than those without co-occurring anxiety disorders (Burns et al. 2005). Further, individuals with co-occurring anxiety disorders and AUD are more likely to experience AUD relapse following treatment than those without co-occurring anxiety (Kushner et al. 2005). These treatment impacts of anxiety are not necessarily due to those with anxiety simply using more alcohol, as one study found that women in treatment for AUD with co-occurring anxiety had lower pretreatment levels of alcohol use than those without anxiety disorders but experienced poorer alcohol outcomes in treatment (Farris et al. 2012). Understanding how to best serve individuals with co-occurring anxiety and AUD is an important research and clinical goal.

Another factor that can add complexity to treatment is rurality. Individuals living in rural areas have historically been underserved in mental health treatment, and a shortage of providers remains a health care crisis in these areas (Larson et al. 2016; Rost et al. 2002; Wang et al. 2006). The Veterans Health Administration (VHA), the largest provider of health care in the USA (Kizer and Dudley 2009), has been a leader in expanding the reach of evidence-based mental health care (Karlin et al. 2012; Karlin and Cross 2014; Karlin et al. 2010; Zeiss and Karlin 2008). However, despite the gains made in mental health treatment access and quality, rural veterans have been underserved with regard to quality mental health care (Mott et al. 2015). Over the past decade, however, the VHA has made marked efforts to improve care in rural areas through video telehealth. In particular, VHA has been at the forefront of video telehealth (VTH) directly to patients’ homes allowing clinicians and patients to engage through a video interface (Lindsay et al. 2017). VA has developed a secure platform, VA Video Connect, that can be over across several devices, and most veterans access their sessions through mobile technologies (i.e., smartphone or tablet) in the private location of their choosing. This technology has helped veterans receive mental health care who would not otherwise have received it (Fletcher et al. 2018; Wynn and Sherrod 2012) and has the capability to deliver evidence-based care effectively (Morland et al. 2019). However, gaps remain, as veterans with substance use disorders (SUD) generally receive less VTH than individuals receiving VTH for other types of mental health disorders (Grubbs et al. 2015). It may be that the addition of AUD to anxiety diagnoses negatively impacts receipt of mental health care via video telehealth, which could further limit rural veterans’ access to the care they need.

As VTH plays more of a prominent role in rural mental health care, it is paramount to understand the impact of this modality of care on service provision and utilization. Prior work has found that, although video telehealth can improve access to care overall, logistical barriers or provider characteristics may limit the types of care provided (Brooks et al. 2013; Gardner et al. 2015). Given the impact of AUD on anxiety treatment and vice versa, and the expanded role of telehealth for rural veterans, it is important to understand current patterns of treatment utilization as they relate to these co-occurring disorders. Our aim in the current study was to use VHA administrative data to examine patterns of video telehealth utilization among rural veterans with anxiety disorders, AUD, and co-occurring disorders.

## Method

Data for this study were obtained through the Corporate Data Warehouse, a large-scale database related to mental and physical health of veterans within the VHA in fiscal years 2016–2019. The Veterans Affairs Information Resource Center monitors the accuracy and validity of the information in the database. This study was approved by the Institutional Review Boards of participating institutions. A national cohort of patients receiving VHA care was identified by selecting veterans who received at least one VTH mental health visit during the data capture period. Demographic data, including rurality (i.e., rural urban commuting area), were obtained through this database.

### Mental Health and AUD Criteria

Anxiety disorder diagnoses were identified by the International Classification of Diseases-10 codes (specific phobia, F40.218, F40.228, F40.230, F40.231, F40.232, F40.233, F40.248, F40.298; social anxiety disorder, F40.10; panic disorder, F41.0; agoraphobia, F40.00; generalized anxiety disorder, F41.1). Individual anxiety disorder diagnoses were combined into one binary anxiety disorder variable. AUD was also defined by presence of ICD-10 codes (F10.10, F10.20). Whether one had a depression diagnosis was included as a covariate in analyses (captured by F32.0, F32.1, F32.2, F32.3, F32.4, F32.5, F32.9, F33.0, F33.1, F33.2, F33.3, F33.41, F33.41, F33.42, F33.9, 300.4, and F34.1).

### Mental Health Services

Current procedural terminology codes were used to identify mental health services accessed by patients during the study time frame. Two groups of mental health visits were created, those linked to VTH visits and those linked to all other visits. Inclusion or exclusion criteria for receiving VTH vary by site to site, depending on availability of services, patient preference, and provider preference; therefore, specific referral information is not captured in the medical record. A total number of mental health visits each year for each type (i.e., VTH or other) were summed to create yearly totals.

### Data Analytic Strategy

The percentage of all mental health visits that was VTH was calculated by dividing the number of VTH visits for each person by his or her number of total mental health visits.

For each participant, we began by examining the total percentage of mental health visits that was VTH over the entire 4-year period. First, we conducted an unadjusted one-way between-groups analysis of variance (ANOVA) to compare the mean percentage of mental health visits that was VTH between the three groups (i.e., anxiety, AUD, and co-occurring AUD and anxiety disorders). Second, we used analysis of covariance (ANCOVA) to repeat this analysis, controlling for the number of fiscal years (i.e., from 1 to 4 years) in which one had any VTH and/or non-VTH mental health visits (adjusted). We then repeated the unadjusted and adjusted analyses controlling for depression diagnosis (where 1 = yes and 0 = no). Any significant effects of group were followed up with pairwise comparisons, using Tukey’s HSD test.

To examine whether there was a significant linear increase in the percentage of mental health visits that was VTH from FY16 to FY19, we employed multilevel linear growth curve models, using PROC MIXED in SAS Version 9.4 (SAS Institute, Inc., Cary, NC). We first examined an unconditional model that included the percentage of mental health visits that was VTH as the outcome and time (FY16 = 0, FY17 = 1, FY18 = 2, and FY19 = 3) as the only fixed predictor. We then examined two conditional models: a main effects model and an interaction model. Both conditional models included the percentage of mental health visits that was VTH as the outcome. The main effects model included both time and group as fixed predictors. The interaction model included time, group, and the interaction between time and group as fixed predictors. A significant interaction between time and group indicates that the linear change over time in the percentage of mental health visits that was VTH differed between groups. Multilevel linear growth curve models were then repeated including depression diagnosis as a covariate. The intercept and slope of time were included as random effects in all multilevel models.

## Results

The final cohort included 10,251 rural veterans. The anxiety-only group comprised 5282 veterans, the AUD-only group comprised 3484 veterans, and the comorbid group comprised 1485 veterans. Demographic information is presented in Table 1.

**Table 1:** Demographic characteristics (*N* = 10,251)

Age, mean (SD)	47.00 (13.67)
Gender, *N* (%)	
Male	8030 (78.33%)
Female	2221 (21.67%)
Race/ethnicity, *N* (%)	
White	8557 (83.47)
Black	914 (8.92)
Asian	55 (0.54)
American Indian/Alaskan Native	178 (1.74)
Native Hawaiian/Other Pacific Islander	86 (0.84)
Hispanic/Latino	475 (4.63)

Unadjusted analyses revealed that the total percentage of mental health visits that was VTH across the 4-year period significantly differed between rural veterans with anxiety disorders only, AUD only, and comorbid anxiety/AUD, *F* (2, 10,248) = 137.36, *p* < .001, *η*^2^ = .03. Post hoc tests revealed that all groups significantly differed from each other. Specifically, veterans with co-occurring anxiety and AUD had a significantly lower percentage of mental health visits that were VTH (*M* = 0.14, *SD* = 0.17) compared with those with anxiety only (*M =* 0.24, *SD* = 0.23) and those with AUD only (*M =* 0.19, *SD* = 0.22) (both ps < 0.001). Furthermore, rural veterans with anxiety disorders only had a significantly greater percentage of mental health visits that were VTH relative to those with AUD only, *p* < 0.001. Analyses that controlled for the number of fiscal years with any mental health visits also revealed a significant difference in the percentage of visits that was VTH between rural veterans with anxiety disorders only, AUD only, and comorbid anxiety/AUD, *F* (3, 10,247) = 130.56, *p* < 0.0001. Post hoc tests again revealed significant differences between all three groups, with rural veterans with co-occurring anxiety and AUD having a significantly lower percentage of mental health visits that were VTH (*M* = 0.16) compared with those with anxiety only (*M* = 0.24) and those with AUD only (*M* = 0.18) (both ps < 0.001). Results of parallel unadjusted and adjusted analyses controlling for depression diagnosis revealed a similar pattern (all groups significantly different from each other at *p* < 0.05).

**Fig. 1:**
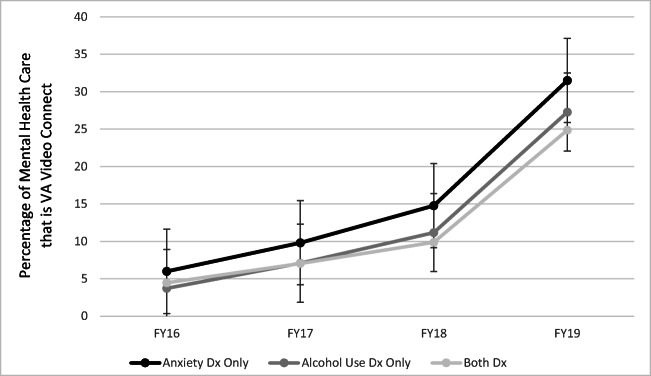
Change from FY16 to FY19 in the total percentage of mental health visits that were video-to-home telehealth, by diagnosis category. Error bars represent standard errors

To examine change over time in the percentage of mental health visits that were VTH, a subset of the cohort (*n =* 4378) was created that consisted of veterans with anxiety disorders only (*n =* 2366), AUD only (*n* = 1671), or both (*n* = 341) in fiscal year 2016. The unconditional linear growth curve model revealed a significant fixed effect of time over the 4-year period, *b* = 0.765, SE = 0.002, *t* (11,000) = 41.36, *p* < 0.0001. Therefore, with each subsequent fiscal year, the percentage of mental health visits that was VTH increased, on average, by 7.65%. Furthermore, the random slope for time was significant (*b* = 0.01, SE = 0.00), *Z* = 16.49, *p* < 0.0001, indicating significant variance between people in linear change in the percentage of mental health visits that was VTH over time. The conditional model revealed significant fixed effects for both time (*b* = 0.08, SE = 0.002, *t* (11,000) = 41.32, *p* < 0.0001) and group (*b* = − 0.02, SE = 0.004, *t* (4062) = − 5.96, *p* < 0.0001). Importantly, the interaction between time and group was also significant (*b* = − 0.008, SE = 0.003, *t* (11,000) = − 2.75, *p* = 0.003; see Fig. 1). Breaking this interaction down revealed a significant effect of time for each group. Specifically, the effect of time was largest for those with an anxiety diagnosis only (*b* = 0.0808, SE = 0.0026, *t* (6001) = 30.65, *p* < 0.0001), followed by those with an AUD diagnosis only (*b* = 0.0731, SE = 0.0029, *t* (4112) = 25.55, *p* < 0.0001), followed by those with both diagnoses (*b* = 0.0611, SE = 0.0058, *t* (932) = 10.67, *p* < 0.0001). With each subsequent fiscal year, the percentage of mental health visits that was VTH increased, on average, by 8.08% for those with an anxiety diagnosis only, by 7.31% for those with an AUD diagnosis only, and by 6.11% for those with both diagnoses. Results retained a similar pattern controlling for depression diagnosis (data not presented).

## Discussion

VTH is an important tool for reaching those who live in rural areas. The findings of the current study suggest that, over the fiscal years of 2016–2019, although telehealth utilization relative to other forms of care grew for veterans with anxiety disorders, similar growth was not observed for those with AUD or co-occurring anxiety disorders and AUD. Interestingly, across years, veterans with co-occurring anxiety and AUD received the least amount of care via VTH. Taken together, these findings suggest that telehealth is not used as much by veterans with co-occurring anxiety and AUD. Given that prior work has found mobile health interventions helpful for reaching individuals with co-occurring mental health and SUD (Ben-Zeev et al. 2014), it may be that specifically targeting this comorbid population through mobile telehealth is needed.

There are several potential explanations for these findings. First, it may be simply that services for AUD are not offered VTH and these veterans received that care in person. However, rural veterans are especially at risk for not receiving SUD care in person (Fortney et al. 1995). Further, rural communities often lack sufficient care for SUD (Fortney and Booth 2001). Given these access challenges in the VHA and the community, telehealth may be one of the primary means of connection to needed mental health and SUD services. Increasing the degree to which SUD care, including for AUD, via VTH could improve access to quality care that may be extremely difficult to access or nonexistent in rural communities. A growing body of work has shown that VTH is a safe and effective modality for delivering care for AUD, including evidence-based psychotherapies (Fletcher et al. 2018; Gros et al. 2013). Although less studied in alcohol users, VTH may also be used for SUD-related medication management, an important tool for treating AUD. Future work would benefit from examining how VTH and other mobile health technologies can be used to address disparities in care.

Interestingly, across years, veterans with co-occurring anxiety and AUD received the least proportion of care via VTH compared with those with anxiety disorders or AUD separately. It may be, then, that current VTH practices do not meet the needs of veterans with co-occurring anxiety and AUD, so they receive more care in person. However, in light of research suggesting that veterans in rural areas are less likely to receive sufficient doses of psychotherapy (Mott et al. 2015) and that SUD services are often unavailable in rural areas (Fortney and Booth 2001), there may be much variability in access and quality of care for comorbid conditions. In the community broadly, telehealth is widely underused for SUD, as it is most often used as a complement to in-person care (Huskamp et al. 2018) which could be a maintaining factor of access challenges. Further, exclusion of telehealth services is often at the discretion of the provider (Cowan et al. 2019), and biases about who may or may not be eligible could prevent those with comorbid diagnoses from receiving care for fear of being too complex to treat via telehealth. However, many patients rate high satisfaction levels with SUD-focused telehealth care (Lin et al. 2019), suggesting that discrepancy between provider and patient beliefs about telehealth is an important area of examination. Also, future work would benefit from closer examination of the types of care available for individuals with these co-occurring disorders in rural areas to determine whether increased VTH utilization could fill gaps in rural mental health care provision for individuals with complex needs for mental health treatment.

The findings must be considered in light of the study’s limitations. One limitation is that the current study used data from the VHA clinical and administrative database. Although this allows robust examination of the health care system database, the diagnoses may not have been assigned on the basis of rigorous assessment methods and errors may have occurred. Also, factors related to using VTH and services for co-occurring disorders are multifaceted and complex; and such clinical decisions are not reflected in the administrative data. Future work would benefit from examining providers’ and patients’ current practices and preferences. Second, the primary outcome of proportion of mental health care received via VTH informs the degree to which VTH penetrates mental health care in rural areas; it does not evaluate care available by other means (e.g., in person, non-VHA care). It is possible that veterans with co-occurring anxiety and AUD are receiving care via other avenues. Further examination of geographic trends and comprehensive evaluation of access to care for these veterans would further inform efforts to use VTH to reach this vulnerable population. Finally, the current study sheds light on a potential disparity among veterans living in rural areas. Further evaluation of access to care and utilization should include examination of differences in care available to veterans in rural and urban areas.

Despite these limitations, the current study begins to fill a gap in our understanding of how veterans in rural areas use VTH and the growth of these services in recent years. As VTH continues to grow as an important tool for reaching underserved rural veterans, attending to trends in care for specific populations can ensure that access to care is improved, even when care needs may be more complex. Understanding how certain clinical populations may experience disparities in telemental health care is important as use of telehealth grows in the face of health care challenges such as the COVID-19 pandemic. For example, given that the VA’s home telehealth platform can be accessed from mobile devices by both patients and providers, VA’s mental health workforce was able to continue to minimize clinical care disruptions even when physical space was limited during pandemic restrictions. Leveraging the success of mobile telehealth to reach underserved populations is an important goal of future work. Comorbid mental health conditions and SUD, including anxiety and AUD, are common; and continuing to innovate service delivery for the individuals that need treatment is an important goal for mental health care systems.
